# The 1990 NIST Scales of Thermal Radiometry

**DOI:** 10.6028/jres.095.050

**Published:** 1990

**Authors:** Klaus D. Mielenz, Robert D. Saunders, Albert C. Parr, Jack J. Hsia

**Affiliations:** National Institute of Standards and Technology, Gaithersburg, MD 20899

**Keywords:** blackbody physics, calibrations, gold point, measurement scales, photometry, pyrometry, radiometry, radiation temperature, temperature scales

## Abstract

Following an absolute NIST measurement of the freezing temperature of gold and the adoption of the International Temperature Scale of 1990 (ITS-90), NIST has adopted new measurement scales for the calibration services based on thermal radiometry. In this paper, the new scales are defined and compared to the ITS-90, and the effects of the scale changes on NIST measurement services in optical pyrometry, radiometry, and photometry are assessed quantitatively. The changes in reported calibration values are within quoted uncertainties, and have resulted in small improvements in accuracy and better consistency with other radiometric scales.

## 1. Introduction

**Figure 1:**
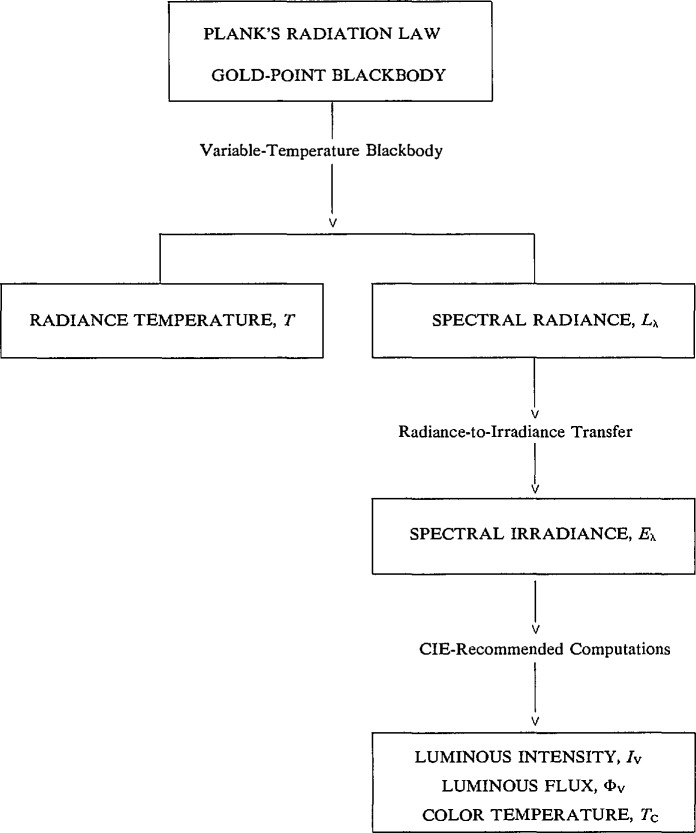
Principal steps in the realization of the NIST measurement scales for thermometry.

The NIST calibration services in thermal radiometry are based on measurement scales derived from blackbody physics. As indicated in figure 1, a blackbody radiator at the temperature of the freezing point of gold and Planck’s radiation law are used to establish the NIST radiance-temperature and spectral-radiance scales by calibrating a variable-temperature blackbody against the gold-point blackbody at 654.6 nm and performing subsequent measurements of spectral-radiance ratios to extrapolate this calibration to extended temperature and spectral ranges. The spectral-radiance scale is then used to derive the NIST scale of spectral irradiance by a radiance-to-irradiance transfer, and hence the NIST scales of luminous intensity, luminous flux, and color temperature are derived by spectral-irradiance calibrations of photometer lamps and computations of these quantities according to the standard procedures established by the Commission International de l’Eclairage (CIE). All of these steps have been documented in NIST publications [1–4]. From 1968 until June 30, 1990, the temperature of the primary blackbody standard used in these scale realizations was that assigned to the freezing point of gold in the International Practical Temperature Scale of 1968 (IPTS-68) [5],

$$T_{68}\left(\text{Au}\right)=1337.58\;\text{K}.$$ (1)

In 1989, an absolute spectroradiometric determination of the temperature of freezing gold was performed at NIST [6] by measuring the spectral radiances of a gold blackbody at wavelengths near 600 nm relative to those of a laser-irradiated integrating sphere which was calibrated with absolute silicon-photodiode detectors and an electrically calibrated radiometer. The result obtained,^1^

$$T_{\text{NIST}}\left(\text{Au}\right)=\left(1337.33\pm 0.34\right)\;\text{K},$$ (2)

is 0.25 K smaller than the IPTS-68 value eq (1) and provided an independent confirmation of measurements by others who had also found a smaller gold-point temperature (see table 3). The NIST gold-point result eq (2) is identical to the value,

$$T_{90}\left(\text{Au}\right)=1337.33\;K,$$ (3)

which is used as one of the fixed points of the new International Temperature Scale of 1990 (ITS-90) [7].

Effective July 1, 1990, the NIST gold point, eq (2), is used instead of the IPTS-68 gold point, eq (1), for the above-mentioned scale realizations, and the limiting uncertainty of the scales is defined in terms of the uncertainty of the NIST gold point. Because the measurement of the NIST gold point employed absolute detector standards, the new 1990 NIST thermal radiometry scales are detector-based scales. Because the NIST and ITS-90 gold-point temperatures, eqs (2) and (3), are identical, these NIST scales are consistent with the ITS-90 definition of radiation temperatures. However, the following differences should be noted:

1. The NIST scales are defined uniquely by the gold point, whereas the radiation temperature range of the ITS-90 is defined in terms of any one of three fixed points: the freezing temperatures of silver, gold, or copper.
2. The NIST scales represent a best estimate of thermodynamic temperature which is consistent with the state of the art of absolute detector radiometry, as practiced at NIST. The ITS-90 is a defined scale which is based on critically evaluated data that were, for the most part, obtained by relative pyrometric measurements performed elsewhere.
3. The NIST scales are used routinely in the entire range (1073–2573 K) in which NIST provides routine measurement services for radiation temperature. The region of the ITS-90 defined by radiation thermometry is limited to temperatures above the silver point (1234.93 K).

Except for small effects on calibration uncertainties, these differences have no practical significance at the present time. NIST personnel will monitor the mutual consistency of the two scales.

In this paper, we describe the new 1990 NIST scales of thermal radiometry, compare them to the ITS-90, and assess the effects of the change on pyrometric, radiometric and photometric calibrations provided by NIST. The calibration services affected are [9]:
SP-250 Test numbersMeasurement service35010C–35030COptical Pyrometers35050C–35060CRadiance Temperature, Ribbon Filament Lamps39010C–39030CSpectral Radiance, Ribbon Filament Lamps39040C–39045CSpectral Irradiance, Quartz-Halogen Lamps39050CSpectral Irradiance, Deuterium Lamps37010C–37070CLuminous Intensity Standards37080C–37130CLuminous Flux Standards37140C–37150CColor Temperature Standards

As will be noted, the changes in reported calibration values are well within the quoted uncertainties of these services but have resulted in small improvements in accuracy and better consistency with other radiometric scales. The NIST calibration services in spectrophotometry (Test Nos. 38010C–38100S) and photodetector response measurements (Test Nos. 39070C–39080S) are not affected by the scale changes described in this paper.

## 2. Radiance Temperature Scale

### 2.1 Definition and Uncertainty of the NIST Scale

As mentioned, the NIST radiation temperature scale is established by measuring the ratio, *r*, of the spectral radiances of a variable-temperature black-body of temperature *T* to that of a gold-point blackbody. The two blackbodies are assumed to be Planckian, so that this ratio can be expressed in the form

$$r=\frac{\text{exp}\left\{c_{2}/\left[n\lambda T_{\text{NIST}}\left(\text{Au}\right)\right]\right\}-1}{\text{exp}\left[c_{2}/\left(n\lambda T_{\text{NIST}}\right)\right]-1}$$ (4a)

where *c*_2_ is the second radiation constant, λ is the air wavelength at which the scale realization is performed (presently 654.6 nm), and *n* is the refractive index of air. Equation (4a) defines the temperature *T*_nist_ in terms of the gold-point temperature eq (2) and a single measurement of the spectral radiance ratio *r* at the discrete wavelength λ. In principle, this temperature is given by

$$\begin{array}{l} \text{exp}\left[c_{2}/\left(n\lambda T_{\text{NIST}}\right)\right]\\ =1+\frac{\text{exp}\left\{c_{2}/\left[n\lambda T_{\text{NIST}}\left(\text{Au}\right)\right]\right\}-1}{r}, \end{array}$$ (4b)

but in practice the scale is realized with spectroradiometers having a finite bandpass and an integral form of eq (4a) is used.

The uncertainty, ∆*T*_nist_(Au), of the NIST value eq (2) with respect to the true thermodynamic gold-point temperature introduces a fundamental limit, ∆*T*_nist_, to the accuracy of the scale at arbitrary temperatures. We can calculate this limiting uncertainty by differentiating eq (4b) with respect to ∆*T*_nist_(Au),

$$\begin{array}{l} \frac{\text{exp}\left[c_{2}/\left(n\lambda T_{\text{NIST}}\right)\right]}{{\left(T_{\text{NIST}}\right)}^{2}}-\frac{\partial T_{\text{NIST}}}{\partial T_{\text{NIST}}\left(\text{Au}\right)}\\ =\frac{\text{exp}\left\{c_{2}/\left[n\lambda T_{\text{NIST}}\left(\text{Au}\right)\right]\right\}}{r{\left[T_{\text{NIST}}\left(\text{Au}\right)\right]}^{2}}, \end{array}$$ (5a)

and then substituting the value of *r* from eq (4a). This gives

$$\begin{array}{l} \Delta T_{\text{NIST}}=\Delta T_{\text{NIST}}\left(\text{Au}\right)\frac{{\left(T_{\text{NIST}}\right)}^{2}}{{\left[T_{\text{NIST}}\left(\text{Au}\right)\right]}^{2}}\\ \times \frac{1-\exp\left[-c_{2}/\left(n\lambda T_{\text{NIST}}\right)\right]}{1-\exp\left\{-c_{2}/\left[n\lambda T_{\text{NIST}}\left(\text{Au}\right)\right]\right\}}. \end{array}$$ (5b)

Numerical values are given in table 1 as a function of temperature and wavelength. They represent the intrinsic uncertainty of the scale and should not be confused with the uncertainty with which the scale can be transferred to calibration customers.

The data in table 1 are indicative of a lack of uniqueness in the scale definition which arises from the fact that the scale realization wavelength λ is not specified. This effect is typical of temperature scales based on optical pyrometry [9] but insignificant for practical purposes. In a hypothetical worst case where the NIST gold point would be 0.34 K too high and the realization wavelength would be changed from 300 to 3000 nm, the scale value at the true temperature 3000 K would only change from 3001.7 to 3001.4 K. The wavelength dependence disappears in the Wien approximation of Planck’s radiation law, when λ*T* ≫ 1 and eqs (4a) and (5b) are reduced to

$$r=\exp\left\{\left(c_{2}/\lambda \right)\left[1/T_{\text{NIST}}\left(\text{Au}\right)-1/T_{\text{NIST}}\right)\right\},$$ (6)

$$\Delta T_{\text{NIST}}=\Delta T_{\text{NIST}}\left(\text{Au}\right)\frac{{\left(T_{\text{NIST}}\right)}^{2}}{{\left[T_{\text{NIST}}\left(\text{Au}\right)\right]}^{2}}.$$ (7)

### 2.2 Changes in Radiation-Temperature Values Reported by NIST

The effect of the 1990 NIST/IPTS-68 gold-point change on NIST radiance temperature calibrations can be quantified by equating the right-hand side of eq (6) with the corresponding expression of the spectral-radiance ratio *r* in terms of the IPTS-68. This leads to

$$1/T_{\text{NIST}}\left(\text{Au}\right)-1/T_{\text{NIST}}=1/T_{68}\left(\text{Au}\right)-1/T_{68},$$ (8a)

or

$$\begin{array}{l} T_{\text{NIST}}-T_{68}=\left[T_{\text{NIST}}\left(\text{Au}\right)-T_{68}\left(\text{Au}\right)\right]\\ \times \left(T_{\text{NIST}}T_{68}\right)/\left[T_{\text{NIST}}\left(\text{Au}\right)T_{68}\left(\text{Au}\right)\right]\\ \approx \left[T_{\text{NIST}}\left(\text{Au}\right)-T_{68}\left(\text{Au}\right)\right]{\left[T_{68}/T_{68}\left(\text{Au}\right)\right]}^{2}. \end{array}$$ (8b)

Using the numerical values given in eqs (1) and (2) we obtain

$$T_{\text{NIST}}\approx T_{68}-\left(0.25K\right){\left(T_{68}\right)}^{2}/{\left(1337.58K\right)}^{2}.$$ (8c)

Numerical examples of this change in reported calibration values are given in table 2. For comparison, the table also lists the uncertainties (3σ) of NIST routine calibrations of radiance temperature on the 1968 and 1990 scales. Hence it may be seen that the changes in value are small and within quoted uncertainties. The slightly lower 1990 uncertainties are due to the fact that the uncertainty of the gold-point realization, taken as 0.4 K in the error budget of the IPTS-68 calibrations, has been replaced by the 0.34 K uncertainty of the NIST gold point, eq (2).

### 2.3 Relation to ITS-90

The radiation-temperature interval of the ITS-90 is defined as follows [7]:

“Above the freezing point of silver the temperature *T*_90_ is defined by the equation

$$\frac{L_{\lambda }\left(T_{90}\right)}{L_{\lambda }\left[T_{90}\left(X\right)\right]}=\frac{\exp\left\{c_{2}/\left[\lambda T_{90}\left(X\right)\right]\right\}-1}{\exp\left[c_{2}/\left(\lambda T_{90}\right)\right]-1},$$

where *T*_90_(X) refers to any of the of the silver [*T*_90_(Ag)= 1234.93 K], the gold [*T*_90_(Au)= 1337.33 K], or the copper [*T*_90_(Cu)= 1357.77 K] freezing points and in which *L*_λ_(*T*_90_) and *L*_λ_[*T*_90_(X)] are the spectral concentrations of the radiance of a black-body at the wavelength (in vacuo) λ at *T*_90_ and at *T*_90_(X) respectively, and c_2_=0.014388 mK.”

This quotation shows that the 1990 NIST and ITS-90 radiation-temperature scales differ in two important respects: range, and uncertainty relative to thermodynamic temperature.

#### 2.3.1 Range

**Figure 2:**
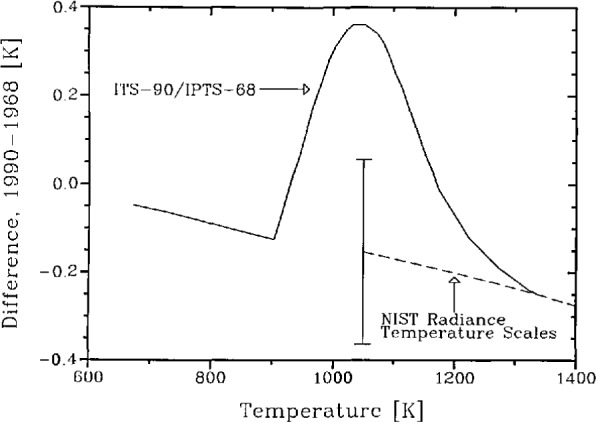
Differences between ITS-90/IPTS-68 (upper curve) and between NIST radiance-temperature measurement based on the 1990 and 1968 gold points (lower curve). The error bar shows the limiting 3σ uncertainty of the 1990 NIST Radiation Thermometry Scale at 1000 K.

In the ITS-90, the Pt-10%Rh/Pt thermocouple has been eliminated as a defining instrument in the 904 to 1337 K interval, and the Pt-resistance- and radiation-thermometry ranges have been extended upwards and downwards to the freezing point of silver, respectively. The differences between the ITS-90 and the IPTS-68 in the temperature interval affected are shown in the upper curve in figure 2. They are believed to represent a substantial improvement over the IPTS-68 with respect to consistency with thermodynamic temperature.

NIST radiation temperature measurements are made in reference to the gold point in the entire range in which these measurements are performed, usually 800–2300 °C (1073–2573 K). This practice was followed even before the IPTS-68 was abrogated. Accordingly, the changes in NIST radiation-temperature measurement services discussed under 2.2 and shown in the lower curve of figure 2 are not the same as the differences between the ITS-90 and the IPTS-68. It is obvious from figure 2 that the adoption of the ITS-90 has removed substantial deficiencies of the IPTS-68 in the thermocouple range, and that NIST measurements performed in this range by contact and radiation thermometry are now in good mutual agreement.

#### 2.3.2 Uncertainty

The redundant definition of the ITS-90 in terms of the silver, gold, and copper points allows alternative scale realizations that have equal status but can give numerically different results. In a footnote to the text of the ITS-90 [7] it is stated that “the *T*_90_ values of the freezing points of silver, gold, and copper are believed to be self consistent to such a degree that the substitution of any one of them in place of one of the other two as the reference temperature *T*_90_(X) will not result in significant differences in the measured values of T_90_.” However, the degree of this self-consistency has not been assessed quantitatively. In the following we present our own assessment, based on a statistical analysis of recent measurements at the silver, gold, and copper points [10–23]. The results of these measurements are listed in table 3. Because all but two of them were performed relative to various reference temperatures, and because these reference temperatures differed with respect to one another and with respect to the ITS-90, we have adjusted these results by applying a correction formula similar to eq (8c),

$$T_{90}=T-\left[T\left(\text{Ref}\right)-T_{90}\left(\text{Ref}\right)\right]{\left[T/T\left(\text{Ref}\right)\right]}^{2},$$ (9)

where *T* and *T* (Ref) denote the published result and the reference temperature used, and *T*_90_ and *T*_90_(Ref) are the corresponding ITS-90 values. The spread in the adjusted temperatures thus obtained is on the order of ±0.1 K (3σ). This figure represents our estimate of the consistency of temperature-scale realizations in the silver-to-copper interval, made relative to alternative ITS-90 fixed points and in different laboratories.

The foregoing statistical analysis does not include an estimate of the accuracy of the reference temperatures of table 3 relative to thermodynamic temperatures. In this respect, the largest component of error is the unexplained difference in the results (*T*−*T*_68_=(−79±6) mK and (−49±20) mK, respectively) of Guildner and Edsinger [24] and Edsinger and Schooley [25] for the departure of the IPTS-68 from thermodynamic temperatures near 729 K, the temperature that served indirectly as the reference for all of the pyrometric measurements in table 3. These results, which do not overlap within their combined 3σ uncertainties, were averaged in the definition of the ITS-90. We estimate that the uncertainty of this average, relative to thermodynamic temperature, is on the order of one half the range of the Guildner-Edsinger and Edsinger-Schooley results (−85 to −29 mK), or ±28 mK. By an expression similar to eq (7), this translates to an uncertainty of ±94 mK at the gold point.

The quadrature combination of the two error uncertainty components mentioned above is ±0.14 K. This number represents our final estimate of the limiting uncertainty, with respect to thermodynamic temperature, of ITS-90 scale realizations near the gold point. It represents an assessment of the combined precision and accuracy within which such scale realizations by primary standards laboratories are consistent with thermodynamic temperature and, as such, is akin to the ±0.34 K uncertainty quoted in eq (2) for the NIST gold point. In spite of this larger uncertainty, the adoption of new NIST scales of thermal radiometry which are independent of the ITS-90 is believed to be justifiable. Their uncertainty constitutes a conservative assessment of current NIST capabilities in absolute radiometry, and their independence will allow future improvements which are in tune with advances in optical radiometry but unencumbered by the state of the art of non-radiometric thermometry at lower temperatures.

## 3. Radiance, Spectral Irradiance, and Photometric Scales

In the derivation of the NIST scales of spectral radiance and irradiance [hereafter denoted by the generalized symbol Q_λ_(T)], the spectral distributions of blackbody sources are expressed by Wien’s equation,

$$Q_{\lambda }\left(T\right)=\left(c_{1}/\lambda^{5}\right)\text{exp}\left(-c_{2}/\lambda T\right),$$ (10)

where *c*_1_ and *c*_2_ are the first and second radiation constants and the refractive index of air is approximated by *n* = 1. The NIST scales of luminous intensity and luminous flux are defined by

$$Q_{v}=K_{m}\int d\lambda V\left(\lambda \right)Q_{\lambda }\left(T\right)$$ (11)

where *Q*_v_ denotes luminous intensity or luminous flux, and *V*(λ) and *K*_m_=683 lm/W, respectively, are the relative spectral luminous efficiency and the maximum luminous efficacy of the 1931 CIE standard observer for photopic vision [26].

The equations governing the effect of the 1990-NIST/IPTS-68 gold-point change on these scales can be derived by differentiation of eq (10) with respect to *T* and substitution of the radiance-temperature scale change eq (8c) into the result obtained. This gives

$$\partial Q_{\lambda }\left(T\right)/\partial T=\left(c_{2}/\lambda T^{2}\right)Q_{\lambda }\left(T\right),$$ (12a)

$$\begin{array}{l} \frac{Q_{\lambda \text{NIST}}-Q_{\lambda ,68}}{Q_{\lambda ,68}}=c_{2}\left[T_{\text{NIST}}\left(\text{Au}\right)-T_{68}\left(\text{Au}\right)\right]\\ ÷\left[\lambda T_{68}^{2}\left(\text{Au}\right)\right]=-2.01\cdot 10^{-3}/\lambda \end{array}$$ (12b)

where λ is expressed in *μ*m. The corresponding photometric scale changes are given by

$$\frac{dQ_{v}}{dT}=K_{m}\int d\lambda V\left(\lambda \right)\frac{\partial Q_{\lambda }\left(T\right)}{\partial T}.$$ (13a)

Hence we obtain, by substitution of eq (12a) into eq (13a),

$$Q_{v,\text{NIST}}=Q_{v,68}\left[1-\left(0.25K\right)q_{v}\right]$$ (13b)

where

$$\begin{array}{l} q_{v}=\frac{c_{2}}{{\left(1337K\right)}^{2}}\\ \times \frac{\int d\lambda \left[V\left(\lambda \right)/\lambda^{6}\right]\exp\left[-c_{2}/\left(\lambda T_{\text{NIST}}\right)\right]}{\int d\lambda \left[V\left(\lambda \right)/\lambda^{5}\right]\exp\left[-c_{2}/\left(\lambda T_{68}\right)\right]}. \end{array}$$ (13c)

Numerical examples of these changes are given in tables 4 and 5, together with the quoted 3σ uncertainties of NIST calibration services. The relative changes in the spectral-radiance and irradiance scales are independent of temperature and inversely proportional to wavelength. The relative changes in the photometric scales were evaluated by numerical integration and exhibit a small, insignificant dependence on temperature.

Although these changes are small, they have helped reconcile small discrepancies that existed in the past. For example, the luminous-intensity data contributed by NIST to a 1985 international intercomparison of photometric base units [27] were the only ones derived from the IPTS-68 gold point. They fell within the spread of the inter-comparison, but were 0.5% higher than the average of the data reported by 14 other national laboratories, all of which had realized the candela with absolute radiometers. When adjusted to the 1990 scale, the NIST data are within approximately 0.1% of the world mean.

The changes in calibration values of color temperature are, in principle, the same as the radiance-temperature changes given by eq (8c) but are too small compared to the quoted uncertainties to warrant a scale change. For example, the change at 2856 K (CIE Source A) would be (−1± 13) K.

## Figures and Tables

**Table 1:** Limiting error (3σ) of the 1990 NIST radiation temperature scale as a function of temperature and scale realization wavelength

Temperature					
Wavelength	1000 K	1500 K	2000 K	3000 K	5000 K
300 nm	0.19 K	0.43 K	0.76 K	1.7 K	4.8 K
655	0.19	0.43	0.76	1.7	4.7
1000	0.19	0.43	0.76	1.7	4.5
1500	0.19	0.43	0.75	1.6	4.0
3000	0.19	0.42	0.71	1.4	3.0

**Table 2:** Changes in reported values and uncertainties (3σ) of NIST radiance temperature calibrations

Value	Change of value	Quoted uncertainty (3σ)	
	*T*_nist_–*T*_68_	1968	1990
800 °C	−0.16 °C	±0.5 °C	+0.5 °C
1100	−0.26	0.6	0.6
1400	−0.39	0.8	0.7
1800	−0.60	1.3	1.2
2300	−0.93	2.0	1.8

**Table 3:** Results of silver-, gold-, and copper-point measurements performed since 1971

Author(s)	Measurement	Reference (K)	Result (K)	Reference adjustment (K)	Adjusted result (K)
Quinn et al. [10]	Ag re Au	1337.58	1235.20	−0.25	1234.987
Bonhoure [11]	Ag re Sb	903.89	1235.16	−0.125	1234.927
Ricolfi and Lanza [12]	Ag re Au	1337.58	1235.20	−0.25	1234.987
Coates and Andrews [13]	Ag re Au	1337.58	1235.22	−0.25	1235.007
Ohtsuka and Bedford [14]	Ag re Cu	1358.03	1235.20	−0.26	1234.985
Jones and Tapping [15]	Ag re Au	1337.58	1235.13	−0.25	1234.917
Andres and Gu [16]	Ag re 630 °C	903.15	1235.21	−0.125	1234.976
Jones and Tapping [17]	Ag re Al	933.452	1235.894	0.021	1234.931
Fischer and Jung [18]	Ag re Al	933.477	1235.927	−0.004	1234.920
Blevin and Brown [19]	Au (absolute)	none	1337.27	none	1337.270
Bonhoure [11]	Au re Sb	903.89	1337.53	−0.125	1337.256
Coslovi et al. [20]	Au re Ag	1235.08	1337.41	−0.15	1337.234
Andrews and Gu [16]	Au re 630 °C	903.15	1337.58	−0.125	1337.306
Jung [21]	Au re Ag	1235.08	1337.45	−0.15	1337.274
Jones and Tapping [17]	Au re Al	933.452	1337.295	0.021	1337.338
Fisher and Jung [18]	Au re Al	933.477	1337.330	−0.004	1337.322
Mielenz et al. [6]	Au (absolute)	none	1337.33	none	1337.330
Righini at al. [22]	Cu re Au	1337.58	1358.02	−0.25	1357.762
Ricolfi and Lanza [12]	Cu re Au	1337.58	1358.02	−0.25	1357.762
Coates and Andrews [23]	Cu re Au	1337.58	1358.04	−0.25	1357.782
Jones and Tapping [14]	Cu re Au	1337.58	1358.04	−0.25	1357.782
Averages and 3σ uncertainties:				Ag	1234.96±0.11
				Au	1337.29±0.11
				Cu	1357.77±0.04

**Table 4:** Changes in reported values and uncertainties (3σ) of NIST spectral-radiance and irradiance calibrations

	Change of value	Quoted uncertainties (3σ)			
		Spectral radiance		Spectral irradiance	
	$\frac{Q_{\lambda ,\text{NIST}}-Q_{\lambda ,68}}{Q_{\lambda ,68}}$	1968	1990	1968	1990
225 nm	−0.89%	±2.1%	±2.0%		
250	−0.80	1.6	1.5	±2.2%	±2.1%
300	−0.67				
350	−0.57	1.2	1.1	1.4	1.3
400	−0.50				
450	−0.45				
500	−0.40				
550	−0.37				
600	−0.34				
654.6	−0.31	0.6	0.6	1.0	1.0
700	−0.28				
800	−0.25				
900	−0.223	0.5	0.5	1.3	1.3
1050	−0.191				
1300	−0.155			1.4	1.4
1600	−0.126	0.4	0.4	1.9	1.9
2000	−0.101			3.3	3.3
2400	−0.084	0.4	0.4	6.5	6.5

**Table 5:** Changes in reported values and uncertainties (3σ) of NIST luminous intensity and flux calibrations

	Change of value	Quoted uncertainties (3σ)			
		Luminous intensity		Luminous flux	
	$\frac{Q_{v\text{.NIST}}-Q_{v.68}}{Q_{\text{v,}68}}$	1968	1990	1968	1990
2000 K	−0.347%				
2400	−0.350				
2600	−0.352				
2856	−0.353	±1.0	±1.0	±1.4	±1.4
3000	−0.354				

## Footnotes

1 All uncertainties quoted in this paper are three-standard-deviation (3σ) estimates.
